# Use of Baclofen Premedication as an Analgesic Adjuvant in Patients Undergoing Percutaneous Nephrolithotripsy: A Placebo-Controlled, Double-Blind Randomized Trial

**DOI:** 10.7759/cureus.64235

**Published:** 2024-07-10

**Authors:** Mark Mandabach, Page Deichmann, Anthony Massoll, Shanna Graves, Dean Assimos, Kyle Wood, Timothy J Ness

**Affiliations:** 1 Anesthesiology and Perioperative Medicine, University of Alabama at Birmingham, Birmingham, USA; 2 Urology, University of Alabama at Birmingham, Birmingham, USA

**Keywords:** urologic surgery, perioperative, gabab receptors, adjuvant, analgesia

## Abstract

Introduction: Baclofen, a clinically available GABA_B_ receptor agonist, produces nonopioid analgesia in multiple models of pain but has had limited studies related to perioperative pain control. The present study seeks to study effects of baclofen on postoperative pain measures and opioid use in adult patients subjected to percutaneous nephrolithotomy (PCNL).

Methods: Using a placebo-controlled, double-blind methodology, a single 10 mg oral dose of baclofen or placebo was given prior to a surgery in 34 patients undergoing PCNL. Standardized intraoperative and postoperative protocols related to opioid use were followed. Use of postoperative opioids in the post-anesthesia care unit and for the first 24 hours following surgery were recorded as were pain scores and other medication use.

Results: There was a significant positive correlation in the use of postoperative opioids in patients who had a preceding history of opioid use. However, there were no significant differences in opioid use which could be attributed to baclofen. There were also no differences in postoperative vital signs, side effects or other medication use.

Conclusions: Analgesic benefits of preoperative baclofen were not observed at the dose employed. Safety of the drug was observed.

## Introduction

Percutaneous nephrolithotomy (PCNL) is an established procedure that is used for removal of large renal calculi, typically > 2 cm in size or in those with complex genitourinary anatomy. It involves placement of a sheath through the skin, subcutaneous tissue and back musculature into a targeted renal calyx to allow for stone fragmentation and removal. A nephrostomy tube is typically inserted into the renal pelvis at the end of the procedure for urine drainage. Patients undergoing PCNL experience acute postoperative pain frequently requiring opioid therapy. The “opioid crisis” has brought attention to the negative physiologic and psychologic side effects of using these agents as well as their potential for misuse/abuse, and addiction. Co-administration of other drugs (e.g., acetaminophen, gabapentin) with opioids has been utilized to increase analgesia and reduce opioid requirements and related side effects in enhanced recovery protocols [1]. New candidates for such adjuvant analgesia include the centrally acting muscle relaxant, baclofen, for which there is an extensive preclinical literature demonstrating that it produces analgesia [2-8]. A GABA derivative and the prototypical GABA_B_ receptor agonist, baclofen has mainly been utilized for its anti-spastic properties. It has been available clinically for over 60 years [9] and in humans, baclofen has had an additional role as an analgesic for cranial nerve-related neuropathic pains such as trigeminal neuralgia [10-13]. In humans, the spinal administration of baclofen has also been demonstrated to have short-term analgesic effects on spinal cord injury-related pain, poststroke pain [14] and, when mixed with a local anesthetic, for postoperative knee pain [15]. However, there are a paucity of studies assessing the effects of systemic baclofen as a perioperative analgesic [16-18]. Gordon et al. [19] observed that it augmented morphine analgesia for postoperative pain management, and Panerai et al. [20] demonstrated baclofen prolonged the analgesic effect of fentanyl administered after surgery.

A primary motivation for performing the present investigation was that baclofen, in both human and preclinical models, has been demonstrated to reduce addictive behaviors. It was our rationale that if we could justify baclofen’s use based on its analgesic potential, then we could plan a larger, repeated dose study that might also examine its effects on subsequent opioid use. Baclofen has been noted to reduce nicotine consumption [21], binge-eating [22], cocaine-craving [23], and alcohol addiction [24] and reviews have noted its beneficial effects in multiple forms of addiction [25]. If similar effects could be noted in humans, there would be huge benefits to be gained for the general population, but this would entail a large sample, prolonged treatment and extensive follow-up. We felt that we first needed to start with a simpler study.

Based on the aforementioned clinical and preclinical reports, baclofen would appear to be an ideal pain-related medication which could maximize analgesia while reducing addictive behaviors. Given the potential of baclofen as a perioperative adjuvant analgesic, we performed the following controlled clinical trial. Our hypothesis was that the co-administration of baclofen with opioids in the PCNL perioperative period would result in improved analgesia and reduced opioid-related side effects.

## Materials and methods

Overview

These studies were approved by the University of Alabama at Birmingham (UAB) Institutional Review Board (IRB; approval April 12, 2019; # IRB-300002466), registered at ClinicalTrials.gov (NCT03720717) and in compliance with the principles of the Declaration of Helsinki. Data was gathered in the UAB Hospitals between April 14, 2019 and March 15, 2023.

Subjects

A total of 40 subjects participated in these studies. The initial six subjects were examined in an open label fashion in order to assess safety factors (there were concerns on the part of the UAB IRB about potential for muscular weakness) and are only described in that data. The subsequent 34 subjects were randomized into two groups: one which received a preoperative oral dose of 10 mg baclofen hydrochloride and the other which received a placebo. Subjects were recruited from patients treated by urologists DA and KW scheduled for unilateral PCNL to remove large kidney stones. Subjects had to be American Society of Anesthesiologist Class 1, 2 or 3 status indicating limited cardiopulmonary or other systemic health issues. Exclusionary criteria per our IRB protocol included a history of cancer and any potential for a difficult airway, but no subjects required exclusion. 

Medications 

Generic baclofen hydrochloride (Endo Pharmaceuticals, Newark DE) was obtained through the UAB Clinical Research Pharmacy and was repackaged into blue gel capsules. Placebo lactose pills were similarly repackaged into identical gel capsules. All drugs were placed into coded, sequentially numbered envelopes such that drug versus placebo presentation occurred in a randomized order. 

PCNL procedure 

After induction of general anesthesia, the patient was placed in the dorsal lithotomy position for cystoscopy retrograde pyelography and placement of an externalized ureteral stent in the targeted renal collecting system. A Foley catheter was placed in all cases. The patient was placed in a prone position and iodinated contrast was injected through the ureteral stent to opacify the collecting system. An 18-gauge needle was placed in a targeted calyx under fluoroscopic guidance. The needle stylet was removed and a guidewire was inserted and manipulated down the ureter and into the bladder. A 1 cm incision incorporating the guidewire tract was made in the skin and subcutaneous tissue. A 30 F dilating balloon was inserted over the guidewire, inflated and a 30 F working sheath was directed over the balloon and into the edge of the targeted calyx. The collecting system was inspected with rigid and flexible nephroscopes and the stone(s) were either removed directly with grasping devices or baskets or fragmented with a rigid dual ultrasonic lithotripsy device or a laser (holmium YAG or thulium). The generated fragments were removed. The whole collecting system was then inspected to confirm that all stones were extracted and an 8 F or 10 F nephrostomy tube was inserted for postoperative urinary drainage. These patients were not thought to be candidates for tubeless PCNL procedures (no nephrostomy tube) or mini-PCNL procedures (utilization of smaller instruments and sheaths), the latter a less invasive approach.

Study protocol

After informed consent was obtained, a medical exam was performed and preoperative medication use was documented. If qualified for participation, subjects were then randomized into treatment versus placebo groups with blinding maintained by use of coded envelopes. Subjects were given their study medication in the preoperative holding area when it was thought to be within 30 minutes of the start of their anesthetic cares. Intraoperative cares were determined by the attending anesthesiologist with the request made by study personnel that fentanyl be the sole opioid utilized and that use be limited to 100 micrograms IV per hour of surgery. In all cases, propofol was utilized as an induction agent and rocuronium was utilized as a muscle relaxant. Anesthesia was maintained with either inhaled sevoflurane or isoflurane and adjusted as deemed necessary by the anesthesia team. Sugammadex was used to reverse any neuromuscular blocking agent in order to allow for a clear assessment of any weakness. Postanesthesia care unit (PACU) arrival pain scores (verbal numerical report: 0=no pain, 10=worst pain ever), arrival hemodynamics and postemergence medication use in the PACU were recorded. Patients were hospitalized overnight and the majority discharged the following day. In hospital analgesic and antiemetic regimens were standardized. Oral morphine equivalent (OME) use was calculated [26] for the first three eight-hour increments; data was extracted from electronic records. Pain scores, vital signs and other medication use were similarly extracted for the first 24 hours of hospitalization.

Statistical analyses

Statistics are presented as the mean ± S.E.M. Repeated measures ANOVAs were performed. Paired t-tests of pre/post measures were used for comparisons when appropriate. Changes in OME use were defined a priori as the primary outcome measured with planned secondary outcome analysis of pain scores, vital signs and other medication use. Missing data (e.g., vital signs) were filled with data from the same patient in the immediately preceding time period. A prestudy power analysis using reductions in OME consumption as the primary outcome had indicated a need to study approximately 80 subjects. Our IRB protocol called for an interim analysis after 40 subjects which included the initial six open label subjects and 34 randomized subjects. At that interim point, a futility analysis [27] using predictive probability assessments led to its early termination.

## Results

Demographics

As apparent from Table 1, preoperative health measures in subjects who received baclofen and those who received placebo treatments were comparable. As preoperative opioid use for the treatment of kidney stone-related pain is common practice in our patient population, an additional stratification of subjects into those with preoperative, daily opioid use of >30 OMEs versus lesser opioid use was performed which again demonstrated no demographic differences.

**Table 1:** Patient Information Data represent Mean+SEM (standard error of the mean) or Incidence as appropriate. See text for stratification criteria. Data analysis ANOVA or Chi Square tests. Sex and ethnicity were self-identified.  ASA (American Society of Anesthesiologists) class was assigned by the attending anesthesiologist. BMI: body mass index; time from the drug to PACU: derived from times for drug consumption and PACU arrival; mg: milligrams; kg: kilograms; m: meters; IV: intravenously; min: minutes; PACU: postanesthesia care unit There were no statistically significant differences between the Baclofen and Placebo groups apart from treatment. There were no statistically significant differences between the Low Opioid and Daily Opioid groups apart from opioid use.

Stratification Category	Baclofen	Placebo	Low Opioid	Daily Opioid
N	17	17	22	12
Age	51+3	46+4	50+4	47+4
ASA Class (2:3)	8:9	8:9	11:11	5:7
Opioid Use (Low:Daily)	12:5	10:7	22:0	12:0
Given Baclofen (Yes:No)	17:0	0:17	12:10	5:7
Sex (Male:Female)	5:12	6:11	8:14	3:9
White:Black:Other	14:3:0	13:4:0	18:4:0	9:3:0
BMI (kg/m^2^)	31.9+1.5	30.9+2.4	31.6+1.7	29.8+2.0
Weight (kg)	91.7+5.7	88.4+6.7	88.4+5.3	89.1+7.0
Time from the drug to PACU (min)	280+16	306+19	281+14	329+25
Midazolam (Y/N) – avg. dose (mg)	(13/4) 1.8+0.3	(15/2) 2.0+0.3	(17/5) 1.7+0.2	(11/1) 2.2+0.3
Intraoperative Fentanyl (mg IV)	222+19	241+24	226+22	231+18

Baclofen effects on analgesic use

**Figure 1:**
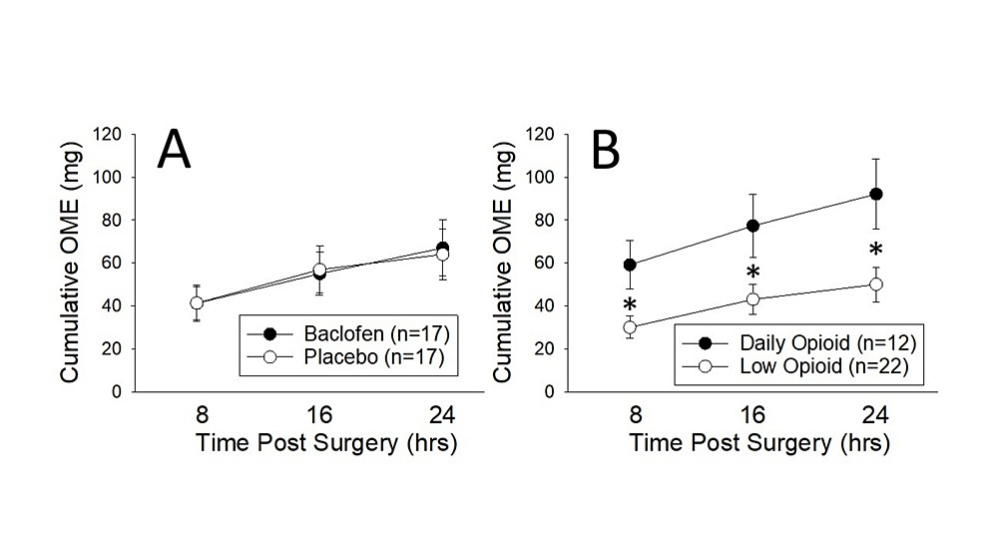
Effect of Baclofen and Preoperative Opioid Use on Analgesic Consumption Cumulative opioid use in eight-hour epochs following surgery was calculated as oral morphine equivalents (OMEs) in study patients stratified according to study treatment (panel A) or according to preoperative opioid use (panel B: see text for complete group descriptions).  No effect of baclofen treatment was noted, but a robust effect of preoperative opioid use was apparent.  * indicates a statistically significant difference between groups with p<0.05.  Data represents mean + SEM of stratified data.

A comparison of opioid use, quantified as OMEs [26] consumed in each eight-hour period for the first 24 hours following surgery, demonstrated no effect of baclofen use on OME use (F1,32 = 0.001; p = 0.984; Figure 1A) or pain scores (F1,32 = 0.020; p = 0.888; Table 2). However, when subjects were stratified according to preoperative daily opioid use, there was a significant difference in OME use (F1,32 = 6.423; p = 0.017; Figure 1B) and pain scores (F1,32 = 28.210; p <0.001; Table 2). There was no statistically significant interaction effect between baclofen analgesic efficacy and preoperative opioid use (F2,2,64 = 2.760; p=0.071 for OMEs; F3,3,96 = 0.544; p=0.654 for pain scores). Notably, the “trend” related to a baclofen interaction with OMEs was in a negative direction indicating that baclofen could have worsened pain control in patients on regular opioids. 

**Table 2:** Outcome Measures Data represents mean+SEM (standard error of the mean) or incidence as appropriate. mg: milligrams; OME indicates oral morphine equivalents [28]. The pain score was a verbal numerical report on a scale of 0-10 where 0: no pain and 10: worst pain. The respiratory rate was measured in breaths per minute.  The heart rate was measured as beats per minute. Systolic and diastolic blood pressures are reported in mm Hg.  See text for explanation of time indicators. There were no statistically significant differences between the Baclofen and Placebo groups on any measure * and ** indicate statistically significant differences from the Low Opioid group with p<0.05 and p<0.01, respectively

Stratification Category	Baclofen	Placebo	Low Opioid	Daily Opioid
8-Hour OME Use (mg)	41+8	41+8	30+5	59+11*
16-Hour OME Use (mg)	55+10	57+11	43+7	77+14*
24-Hour OME Use (mg)	67+13	64+12	50+8	92+16*
Initial Postop Pain Score (0-10)	2.6+0.8	2.8+0.9	1.1+0.4	5.5+1.1**
Initial Respiratory Rate	17+1	16+1	16+1	16+1
Initial Heart Rate	90+3	93+4	93+3	88+5
Initial Systolic Blood Pressure	129+4	127+4	127+4	128+3
Initial Diastolic Blood Pressure	71+3	71+4	70+3	72+4
8-Hour Postop Pain Score (0-10)	2.7+0.8	3.0+0.8	1.6+0.6	4.7+0.9*
8-Hour Respiratory Rate	21+5	16+1	20+4	16+2
8-Hour Heart Rate	81+5	90+4	86+4	84+5
8-Hour Systolic Blood Pressure	126+4	117+4	122+4	118+5
8-Hour Diastolic Blood Pressure	72+4	67+3	70+3	69+4
16-Hour Postop Pain Score (0-10)	2.8+0.8	2.7+0.9	1.4+0.7	5.3+0.9**
16-Hour Respiratory Rate	18+1	17+1	18+1	17+1
16-Hour Heart Rate	83+3	88+5	87+3	84+7
16-Hour Systolic Blood Pressure	127+4	115+4	123+3	115+5
16-Hour Diastolic Blood Pressure	73+4	66+4	69+3	68+3
24-Hour Postop Pain Score (0-10)	2.5+0.8	2.8+0.9	1.2+0.5	5.5+0.9**
24-Hour Respiratory Rate	17+1	16+1	17+1	16+1
24-Hour Heart Rate	81+3	89+4	86+3	84+6
24-Hour Systolic Blood Pressure	129+5	119+4	126+3	119+6
24-Hour Diastolic Blood Pressure	72+2	69+3	70+3	71+3
24-Hour Antiemetic (Yes:No)	4:13	6:11	4:18	6:6
24-Hour Anxiolytic (Yes:No)	1:16	0:17	0:22	1:11

Baclofen side effects

Vital signs, antiemetic use and other medication use were comparable between groups (Table 2). There were no reports of weakness, respiratory compromise or confusion in any of the groups. One of the open-label subjects suffered sufficient blood loss associated with the surgical procedure that they required transfusion and admission to an intensive care unit following the procedure. That patient demonstrated no muscle weakness or mental status changes. No other significant adverse events occurred.

## Discussion

The most important finding of the present study was that the nonopioid agent baclofen, when used as a single dose, perioperative adjuvant in patients subjected to PCNL, had no significant effects on pain scores or on subsequent opioid use at the dose employed (10 mg p.o.). This result was surprising, given the theoretical potential for benefit. As previously described, both preclinical [2-8] and clinical [9-20] studies predicted an analgesic effect. Subsequent studies may be able to observe analgesic effects if higher doses and/or repeated dosing of the drug are used in the perioperative period. Consideration could also be given to potential parenteral administration of the baclofen as this route would have more reliable pharmacokinetics and would allow more precise titration of the drug. Spinal intrathecal delivery is also possible but would require an invasive procedure. Notably, the dose of baclofen utilized was a typical clinical dose [9] which was well tolerated and was not associated with unacceptable side effects and so use of even higher and repeated doses appears feasible. That said, higher doses have been associated with the development of postoperative delirium and other side effects [18]. Our use of a single, preoperative 10 mg dose was based on significant caution on our part in relation to the potential for muscle weakness and respiratory compromise in the perioperative setting which were not observed. 

Basic science studies suggest that GABA_B_ receptor activation may interfere with some endogenous pain control systems [28] and so there is potential for complex functional interactions that may preclude general use of the drug in this setting. Other candidates for nonopioid augmentation of analgesic effects that might have made a baclofen effect more apparent include ketamine, anti-inflammatories, antidepressants, anticonvulsants, and some antiarrhythmic agents but use of such agents will require similar, cautious controlled trials to assess analgesic potential. 

The surgical procedure studied here (percutaneous nephrolithotomy) may or may not have been an optimal choice for study. Since muscular back spasms can occur after this procedure, we hypothesized that an extra benefit of the baclofen may have been noted due to its anti-spastic effects. However, this was not the case. There was also great variability in individual’s postoperative opioid use in relation to this surgery which would also likely have reduced the ability to observe an effect. Studies that have identified perioperative benefits of baclofen have been ones with limited variability such as third molar extractions [19]. We did not limit the study to opioid-naïve subjects which also added to variability. However, a post hoc analysis did not suggest that pre-operative opioid use affected the efficacy of the baclofen, just the efficacy of the subsequent opioids. The observation of elevated OME consumption in potentially opioid tolerant patients gives validation to the methodology of the study. Regional anesthetic strategies have been proposed to limit postoperative pain after PCNL [29,30]. However, while these blocks appear to be beneficial, they are invasive. None of these other approaches were utilized in this study.

## Conclusions

In summary, the compound baclofen, a nonopioid drug, as a single preoperative dose of 10 mg p.o. was demonstrated to have no benefit on postoperative pain control in patients who underwent PCNL but was without observed toxicity. A higher and/or repeated dose of baclofen might have had a different outcome and so should be utilized if additional clinical trials are entertained. Likewise, parenteral or intrathecal drug delivery also has potential for greater analgesic benefits but would require continued assessment of toxicities. 
